# Management of Neonatal Respiratory Distress Syndrome Employing ACoRN Respiratory Sequence Protocol versus Early Nasal Continuous Positive Airway Pressure Protocol

**Published:** 2014-01-24

**Authors:** Pedram Niknafs, Asadallah Faghani, Seyed-Abolfazl Afjeh, Mehdi Moradinazer, Bahareh Bahman-Bijari

**Affiliations:** 1 Afzalipour Medical Center; 2 Mahdieh Medical Center, Shahid Beheshti University of Medical Sciences, Tehran, IR Iran; 3 Research Center for Modeling in Health, Institute for Futures Studies in Health,Kerman University of Medical Sciences, Kerman

**Keywords:** Respiratory Distress Syndrome, Neonates, Outcomes, Management, ACoRN

## Abstract

***Objective:*** Respiratory distress syndrome (RDS) is a common cause of respiratory distress in premature infants. This study was designed to evaluate two different RDS treatment protocols by comparing the outcomes.

***Methods:*** This study was a double center cross sectional study performed from June to December 2012. During that period, 386 neonates with RDS were hospitalized and treated according to two different therapeutic protocols so-called Acute Care of at-Risk Newborns (ACoRN) respiratory sequence protocol (group I) and Early Nasal Continuous Positive Airway Pressure (E-NCPAP) protocol (group II). The variables and main outcomes of this study were gestational age, birth weight, bronchopulmonary dysplasia (BPD), pulmonary hemorrhage (PH), intraventricular hemorrhage (IVH), air leak and mortality rate (MR).

***Findings*** ***:*** Out of 386 infants, 202 infants were in group I (male 60.4%, female 39.6%, mean gestational age 31^6/7 ^weeks, mean birth weight=1688 grams) and group II included 184 infants (male 61.4%, female 38.6%, mean gestational age 32 weeks, mean birth weight 1787 grams), *P*= 0.07. The ratios of BPD of group I to group II and PH of group I to group two were not significant (*P*=0.63 and *P*=0.84, respectively). Air leak ratio in group I was higher than in group II (*P*=0.001). Although IVH ratio in group II was higher than in group I (*P*=0.01), grade III and IV IVH was higher in group I (30% vs. 4.6%). In case of MR, it was higher in group I than in group II (*P*=0.001).

***Conclusion:*** According to the findings the incidence of air leak, grade III and IV IVH and MR was less common in E-NCPAP protocol, so it may show the effectiveness of this protocol. The authors suggest that more researches are needed for more accurate results.

## Introduction

Respiratory distress is the most common cause for admission to the Neonatal Intensive Care Unit (NICU). Clinical presentation of respiratory distress in neonates includes tachypnea, poor feeding, nasal flaring, grunting, cyanosis, intercostals retraction and reduction of respiratory sounds in lungs auscultation. Respiratory distress is seen in 6%-7% of newborns, the highest incidence rate being related to preterm neonates followed by post term and term infants^[^^1^^]^. 

 Respiratory distress syndrome (RDS) is a common cause of respiratory distress in preterm neonates. The pathophysiology of RDS is surfactant deficiency of the lungs of infants. There is a reverse relationship between the incidence of RDS and gestational age ^[^^2^^]^ or birth weight, as 44% of newborns weighing between 501 to 1500 grams develop RDS^[^^3^^]^. Respiratory distress, chest radiographic findings (bilateral reticulogranular or ground glass and air bronchogram patterns) and acidosis (respiratory and/or metabolic) are the main clinical and paraclinical diagnostic clues. The basis of treatment for patients who are suffering from RDS is the use of surfactant and respiratory support i.e. Mechanical Ventilation (MV) and Nasal Continuous Positive Airway Pressure (NCPAP) ^[^^4^^]^. It is also important to note that antenatal corticosteroid therapy not only reduces significantly mortality and morbidity rate in premature neonates particularly neonates with the gestational age of 24-34 weeks, but also decreases the treatment expenses enormously^[^^5^^]^. In the course of RDS treatment some short term or long term complications such as air leak, intraventricular hemorrhage (IVH), broncho pulmonary dysplasia (BPD) and retinopathy of prematurity (ROP) can be seen in patients. This complications and also mortality rate (MR) and variables such as duration of hospital stay and cost of treatment can be influenced by treatment protocols. 

 The aim of this study was to verify the effectiveness of treatment protocols by comparing the outcomes of RDS treatment with two different therapeutic protocols which we called Acute Care of at-Risk Newborns (ACoRN) respiratory sequence protocol (group I) and Early Nasal Continuous Positive Airway Pressure (E-NCPAP) protocol (group II).

## Subjects and Methods

This was a double center cross sectional study to compare the RDS outcomes between two groups of patients being treated in two neonatal intensive care units (NICU) with different therapeutic protocols in a six-month period (from June 21^st^ 2012 to December 20^th^ 2012). Patients’ assessments were done by fellowship in neonatology.

 The including factor was inborn infants who suffered from RDS and the excluding factors were outborn infants and also the cases that suffered from RDS accompanied by asphyxia, congenital anomalies, congenital sepsis, congenital pneumonia and meconium aspiration syndrome. 

 The first protocol was ACoRN respiratory sequence protocol (group I) which took place in Afzalipour Medical Center in Kerman affiliated with Kerman University of Medical Sciences. Afzalipour Medical Center NICU has 16 level III and 10 level II beds. For performing the protocol ACoRN respiratory sequence was used^[^^6^^]^. 

 ACoRN is a sequential and algorithmic framework to assess, monitor, intervene, and manage at-risk and critical newborns. ACoRN respiratory sequence protocol uses a scoring system (Table 1) which is derived from the clinical judgment of physicians and gestational age of the newborn. This scoring system allows the physician to assess the patients’ clinical symptoms according to the given score (respiratory score <5, 5-8, >8 for mild, moderate and severe respiratory distress respectively) in order to choose the appropriate respiratory support (oxygen therapy by hood, NCPAP or MV)^[^^6^^]^. Therefore when a neonate was admitted with respiratory distress, in the case of using oxyhood (mild respiratory distress or respiratory score <5), an amount of FiO_2_ with 40% oxygen concentration and warm and humid flow of 5 liters per minute was started.

**Table 1:** ACoRN Respiratory Score

**Score**	**0**	**1**	**2**
**Respiratory rate**	40 to 60 / minute	60 to 80 / minute	>80 / minute
**Oxygen requirement** ^1^	None	≤50%	>50%
**Retractions**	None	Mild to moderate	Severe
**Grunting**	None	With stimulation	Continuous at rest
**Breath sounds on auscultation**	Easily heard throughout	Decreased	Barely heard
**Prematurity**	>34 weeks	30 to 34 weeks	<30 weeks

A baby receiving oxygen prior to the setup of an oxygen analyzer should be assigned a score of “1”. (Derived from reference 6)

For keeping the patient’s blood oxygen saturation at the level of 85%-95%, the amount of FiO_2 _could be increased to 60%. If the patient’s respiratory score was in the range of 5-8, respiratory support would be established as NCPAP (through bubble CPAP or ventilator CPAP) with the maximum pressure of 6 cm H_2_O and for maintaining the patient’s blood oxygen saturation at the level of 85%-95% the amount of FiO_2 _could be increased to 100%. If the respiratory score was more than 8, the patient would be intubated and treated with mechanical ventilation (MV) using the Assist/Control (A/C) mode or the Synchronized Intermittent Mandatory Ventilation (SIMV) mode. 

 It was necessary to use NCPAP respiratory support instead of oxyhood if Arterial Blood Gas (ABG) result was the following amounts: PaO_2 _<50 mm Hg and pH>7.2 and PaCO_2_<60 mm Hg and it was necessary to use ventilator respiratory support instead of NCPAP if ABG result was the following amounts: PaO_2_<50 mm Hg and pH<7.2 and/or PaCO_2_>60 mm Hg. 

 It should be noted that during treatment if according to clinical and paraclinical judgment, surfactant instillation was necessary, the treatment would be performed with the INtubation, SURfactant administration, rapid Extubation to NCPAP (INSURE) method and in the case of respiratory failure probability the patient would be intubated and treated by ventilator. 

**Fig. 1:**
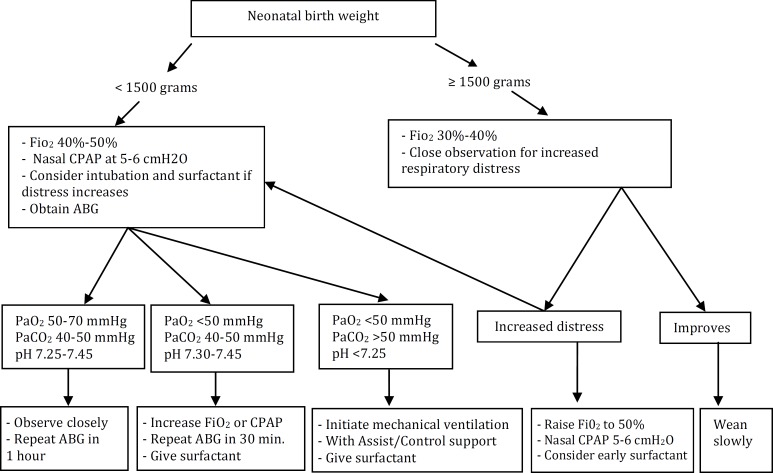
Early-NCPAP protocol approach (group II).

 In data collection phase of group I a total of 253 inborn neonates with respiratory distress were admitted to NICU but because of excluding factors, only 202 patients were included in this group. The second protocol was Early Nasal Continuous Positive Airway Pressure protocol (group II), which was conducted in Mahdieh Medical Center in Tehran affiliated with Shahid Beheshti University of Medical Sciences. The NICU of Mahdieh Medical Center has 33 level III and 15 level II beds. In that protocol, neonates admitted with RDS were divided in two groups based on their birth weight: >1500 grams and ≤1500 grams, and were treated and received respiratory supports according to Fig 1^[^^7^^]^. During the study period, 258 inborn neonates with respiratory distress were admitted to group II NICU but because of excluding factors, only 184 cases were included in this group.

NCPAP: Nasal continuous positive air way pressure, ABG: Arterial blood gas (Derived from reference 7)

**Table 2:** Demographic variables in group I and group II

**Variable**	**Group I** ^†^	**Group II** ^‡^	***P.*** ** value**
**Frequency**	202	184	0.08
**Gestational age (SD) **	31 wks and 6 days (±2 wks and 4 days)	32 wk (±1 day)	0.7
**Birth weight (SD) grams**	1688 (±532)	1787 (±723)	0.12

† Acute Care of at-Risk Newborns respiratory sequence protocol group,

‡ Early Nasal Continuous Positive Airway Pressure protocol group

The primary end point in this study was recovery and hospital discharge or death.

 The variables of this study were gestational age (GE), gender, birth weight, duration of oxygen therapy by hood, duration of NCPAP therapy, duration of ventilator therapy, treatment with surfactant, air leak, intraventricular hemorrhage (IVH), pulmonary hemorrhage, retinopathy of prematurity (ROP), mortality rate, length of hospital stay and the costs of treatment. 

 Finally, the required data which were collected from pre-prepared questionnaires were analyzed with the SPSS 19 software. For the data analysis, in addition to data description the chi-square test was used for ratio comparison, and for comparing the means between the study groups, the two way analysis of variance (Two Way-ANOVA) was used. In this study the significance level which was considered for all tests was *P*<0.05.

 This study was approved in ethics committee of Kerman University of Medical sciences (ethic code number, 283/91/K).

## Findings

In our study RDS was the most common reason for neonates’ admission due to respiratory distress in NICUs which included 74% and 65.8% of neonates <34 weeks in group I and group II protocols respectively (*P*=0.07). In other words, 43% and 40% of neonates weighing less than 1500 grams in the two above-mentioned protocols suffered from RDS respectively.

 From 202 neonates in group I, 60.4% cases were boys and 39.6% cases girls and from 184 neonates in group II, 61.4% cases were boys and 38.6% girls (*P*=0.9). Table 2 shows demographic variables in group I and group II. Intraventricular hemorrhage (IVH) was seen in 10 cases (approximately 5% of patients) in group I, 70% being neonates <30 weeks, and 30% were neonates with gestaional age of 30 to 34 weeks (*P*=0.001). Thirty percent of cases whose gestational age was <30 weeks suffered from grade III and IV IVH. Neonates >34 weeks in this protocol had no IVH. IVH occurred in 22 cases (approximately 12% of patients) in group II 54.5% of which were neonates <30 weeks, 36.4% between 30-34 weeks and 9.1% neonates >34 weeks (*P*=0.01). Grade III IVH was seen in 4.6% cases which involved neonates <30 weeks (Grade IV IVH was not reported in this protocol). 

 Detailed information was not obtained for retinopathy of prematurity (ROP) in group I. In group II ROP occurred in 24 cases (approximately 13% patients), 70.8% being neonates <30 weeks, 25% 30-34 weeks and 4.2% >34 weeks (*P*=0.001). Only 16.6% of ROP cases were stage III and IV (41.7% stage I and 41.7% stage II). Comparison of variables and outcomes of the two protocols are summarized in Tables 3, 4 and 5.

## Discussion

**Air leak: **The incidence of this complication was more common in group I than in group II (Table 5). The incidence of this morbidity in article reviews was reported about 6.3% among very low birth weight (VLBW) infants^[^^8^^]^, therefore considering that the usage of surfactant in those two protocols was not significant (*P*=0.96). It seems that group II got better results in reducing this complication in patients with RDS.

**IVH: **The incidence of this morbidity in different studies and different ages are variable, but clearly this morbidity has a reverse ratio with gestational age and birth weight. In our study the incidence of this morbidity in group I was lower than in group II (Table5), but it is important to mention that the incidence of grade ≥III IVH in group II was 4.6% and in group I about 30%.

**Table 3:** Comparison of treatment duration and costs of treatment in three gestational age groups in two protocols

**Variable**	**Protocol**	**Gestational age**	**Mean (SD)**	***P *** **value**
**Duration of oxyhood treatment (day)**	Group I	<30 weeks	4.6 (8.2)	0.3
		30-34 weeks	3.5 (4.8)	
		>34 weeks	2.8 (3)	
	Group II	<30 weeks	6.5 (9.5)	0.001
		30-34 weeks	3.7 (4.8)	
		>34 weeks	2 (1.9)	
**Duration of NCPAP treatment (Day)**	Group I	<30 weeks	2.8 (2.9)	0.04
		30-34 weeks	1.8 (2.2)	
		>34 weeks	1.9 (1.4)	
	Group II	<30 weeks	2.8 (8.5)	0.001
		30-34 weeks	2.7 (3.4)	
		>34 weeks	1.7 (1.5)	
**Duration of Ventilator treatment (Day)**	Group I	<30 weeks	3.6 (5.9)	0.001
		30-34 weeks	1.3 (2.5)	
		>34 weeks	0.5 (1.3)	
	Group II	<30 weeks	4.3 (10.2)	0.001
		30-34 weeks	0.7 (2)	
		>34 weeks	0.6 (1.5)	
**Costs of treatment (US $)**	Group I	<30 weeks	1200.00 (977.00)	0.002
		30-34 weeks	809.00 (705.00)	
		>34 weeks	716.00 (399.00)	
	Group II	<30 weeks	3578.00 (2862.00)	0.001
		30-34 weeks	1886.00 (1334.00)	
		>34 weeks	902.00 (531.00)	
**Duration of hospital stay (Day)**	Group I	<30 weeks	15.4 (13.1)	0.002
		30-34 weeks	11.4 (8.3)	
		>34 weeks	8 (2.7)	
	Group II	<30 weeks	33.6 (28.5)	0.001
		30-34 weeks	18.4 (13.1)	
		>34 weeks	8.9 (4.8)	

Group I: Acute Care of at-Risk Newborns respiratory sequence protocol group; Group II: Early NCPAP protocol group; NCPAP: Nasal Continuous Positive Airway Pressure

In literature review IVH morbidity incidence was about 5.3% in neonates ˂37 weeks^[^^9^^]^, and about 25% in neonates between 501 and 1500 grams^[^^10^^]^.

**BPD: **This morbidity was proposed more than 40

years ago and it is one of the most serious and important problems in very premature neonates^[^^11^^]^. The overall incidence of this morbidity in our study in group I and group II was 3% and 7.6%, respectively (Table 5). In literature review BPD incidence was reported about 25% in neonates less than 1500 grams^[^^12^^]^. 

**Table 4:** Comparison of treatment days, costs and hospital stay variables in two protocols

**Variable**	**Protocol**	**Mean (SD)**	***P*** ** value**
**Duration of oxyhood treatment (day)**	Group I	3.6 (5.5)	0.5
	Group II	4 (6.3)	
**Duration of NCPAP treatment (day)**	Group I	2.1 (1.4)	0.001
	Group II	3.3 (0.3)	
**Duration of ventilator treatment (day)**	Group I	1.6 (0.42)	0.8
	Group II	1.6 (0.37)	
**Duration of hospital stay (days)**	Group I	10.9 (1.1)	0.001
	Group II	18.2 (1.3)	
**Costs of treatment (US $)**	Group I	915.00 (113.00)	0.001
	Group II	1906.00 (98.00)	

Group I: Acute Care of at-Risk Newborns respiratory sequence protocol group; Group II: Early NCPAP protocol group; NCPAP: Nasal Continuous Positive Airway Pressure

**Table 5:** Comparison of treatment outcomes, mortality rate and use of surfactant in two protocols

**Variable**	**Protocol**	**n (%)**	***P *** **value**
**Air leak**	Group I	33 (16.8)	0.001
	Group II	7 (3.8)	
**Bronchopulmonary dysplasia**	Group I	6 (3)	0.6
	Group II	14 (7.6)	
**Intraventricular hemorrhage**	Group I	10 (5)	0.01
	Group II	22 (11.9)	
**Pulmonary hemorrhage**	Group I	15 (7.4)	0.8
	Group II	12 (7)	
**Mortality rate**	Group I	52 (25.7)	0.001
	Group II	21 (11.4)	
**Use of surfactant**	Group I	89 (44.1)	0.9
	Group II	65 (35.3)	

Group I: Acute Care of at-Risk Newborns respiratory sequence protocol group; Group II: Early Nasal Continuous Positive Airway Pressure protocol group

The lower BPD incidence in group I than in group II could be due to higher mortality rate in group I.

**Pulmonary hemorrhage (PH): **The incidence of PH in our study in group I and group II occurred in 7.4% and 7%, respectively (*P*=0.84) (Table5). This complication is very important because it increases the mortality rate especially in VLBW infants. The incidence of PH in VLBW neonates was 5.7% in review article^[^^13^^]^. 

**Retinopathy of prematurity (ROP): **ROP morbidity is developmental disorder of retinal vessels in premature neonates and it is one of the causes of blindness in 10% of children in developed countries^[^^14^^,^^15^^]^. Unfortunately, in group I due to lack of an ophthalmologic referral clinic in the hospital, no written records were obtained and patients were seen by ophthalmologist in private clinics outside the hospital. These cases were followed up by telephone calls with the family and we would like to draw the attention of the authorities to this matter. Although there was no case that required surgery after the hospital discharge during the four months of telephone follow-up in group I, there was no statistics about the incidences of different stages in this protocol. In group II from the 24 cases with ROP, 41.7% were stage I, 41.7% stage II and 16.6% stage III and above. All of the neonates in ROP stage III and above underwent surgical treatment. ROP incidence in group II was relatively consistent with the other studies. According to the study was conducted in Singapore, 29.2% of neonates less than 1500 grams suffered from ROP, 49% of whom were in stage I, 24% in stage II and 27% in stage III and above. From the patients in the last stage 62.2% underwent laser therapy or surgical

treatment with cryotherapy^[^^16^^]^.

**Mortality rate (MR): **Evidence suggests a significant decrease in deaths due to RDS in the past four decades around the world especially in developed countries, yet this reduction is not the same in all countries. As mentioned in result section, MR was higher in the group I than in group II (25.7 vs 11.4) (Table 5). To determine whether the treatment protocol caused this significant difference or other factors were also involved, requires further studies. Although not much research on RDS specific mortality rate is available in less developed countries, a study which took place in South America reported 25.9% death due to RDS^[^^17^^]^. 

**Duration of hospital stay: **In our study, the mean hospital stay for all patients in group II was higher [18.2(±1.3) days] than in group I [10.9(±1.1) days] (Table 4). As it can be seen in result section (Table3), the mean duration of hospital stay in neonates over 34 weeks in groups I and II did not have an obvious difference (8 days vs 8.9 days), but this difference was significant in <30 weeks gestational age (15.4 days vs 33.6 days in groups I and II, respectively) and we believe that NICUs’ policy of discharge weight made that difference (*P*=0.001). In a study conducted in California, USA, the average hospital stay for low birth weight neonates was reported between 6.2 days and 68.1 days variable^[^^18^^]^. 

**Costs of treatment:** Mean costs of treatment for hospitalized patients was higher in group II than in group I (Table 3 and 4). Costs of treatment have a direct relation with duration of hospital stay. According to the study which was published in USA in 2007 (however, these costs were reported for neonates’ treatment in 1999-2000), mean costs of treatment for neonates of 24-31 weeks gestational age was $5393.00 and for neonates 32-36 weeks gestational age $1575.00^[^^19^^]^.

## Conclusion

The incidence of RDS in our study was almost identical in two groups. According to the findings the incidence of air leak, grade III and IV IVH and MR was less common in E-NCPAP protocol, so it may show the effectiveness of that protocol. The incidence of BPD morbidity in group I was lower than that in review articles. The incidence of pulmonary hemorrhage in both groups was higher than that in developed countries; therefore this fatal complication needs more consideration for prevention. It is suggested to evaluate duration of hospital stay and costs of treatment, optimal discharge weight also should be one of the crucial variables in future studies. 
